# LL-37 Inhibits EV71 Infection by Upregulating STAC via the EGFR-ERK Signaling Pathway

**DOI:** 10.3390/v18040442

**Published:** 2026-04-07

**Authors:** Jiaqi Zhang, Hanlin Zhang, Yi Chen, Hanfei Liu, Shuhuang Peng, Jiwei Zhao, Zhe Luan, Yujian Zhang, Meng Dong, Wanzhu Jin, Gang Sun

**Affiliations:** 1 Medical School of Chinese People’s Liberation Army, Beijing 100853, China; jennyzjq@163.com (J.Z.); cydoctor26@163.com (Y.C.); zhaojiwei1005@163.com (J.Z.); 2 Department of Gastroenterology and Hepatology, The First Medical Center, Chinese PLA General Hospital, No. 28 Fuxing Road, Haidian District, Beijing 100853, China; luanzhe@foxmail.com; 3 State Key Laboratory of Animal Biodiversity Conservation and Integrated Pest Management, Institute of Zoology, Chinese Academy of Sciences, Beijing 100101, China; zhanghanlin@ioz.ac.cn (H.Z.); zhangyujian2023@ioz.ac.cn (Y.Z.); dongmeng@ioz.ac.cn (M.D.); 4 College of Life Sciences, University of Chinese Academy of Sciences, Beijing 101408, China

**Keywords:** cathelicidin, EV71, host defense peptide, STAC

## Abstract

LL-37, a 37-amino acid human-derived antimicrobial peptide, was shown in our earlier clinical study to shorten the negative conversion time of the Omicron BA.5.1.3 variant of SARS-CoV-2. In this work, we investigated the broad mechanism of LL-37 by examining its inhibitory effect on non-enveloped virus *Enterovirus 71* (EV71). LL-37 treatment dose-dependently reduced EV71 viral RNA abundance, suppressed virus-encoded protein expression, and decreased infectious titers, acting predominantly at a post-entry stage of the viral life cycle. Transcriptomic analysis revealed that the SH3 and cysteine-rich domain protein (Stac) was uniquely upregulated by LL-37 irrespective of EV71 infection. Short hairpin RNA (shRNA)-mediated Stac silencing significantly enhanced EV71 infection, while Stac overexpression markedly reduced it. Furthermore, we found that LL-37 activates the EGFR–ERK signaling pathway, leading to time-dependent upregulation of Stac expression. These findings uncover a novel host-directed mechanism by which LL-37 combats EV71 infection and suggests a potential therapeutic use of LL-37 against non-enveloped viral disease.

## 1. Introduction

LL-37, the sole cathelicidin in humans, is a 37-amino-acid, amphipathic cationic antimicrobial peptide [1]. Its precursor is secreted by various epithelial cells and immune cells. LL-37 is constitutively expressed in bodily compartments exposed to external environments, such as the airway, gut, and urinary tract [2,3]. LL-37 is critical in combating microbial infections, modulating host immune responses, and recruiting immune cells [4]. These functions are primarily mediated through the interaction with host cell surface receptors, such as G protein-coupled receptors (GPCRs), receptor tyrosine kinases (RTKs), and purinergic receptors [5].

LL-37 exhibits antiviral functions against multiple enveloped and non-enveloped viruses. Recently, we have shown that oral recombinant LL-37 Lactococcus lactis significantly shortens the negative conversion time of the enveloped Omicron BA.5.1.3 variant of SARS-CoV-2 [6]. Similarly, exosome-loaded LL-37 significantly reduced Zika Virus (ZIKV) infection in vitro and attenuated ZIKV-induced tissue damage in vivo [7]. LL-37 reduced Rhinovirus (RV) replication, and its level in bronchoalveolar lavage of children with cystic fibrosis was inversely correlated with RV load [8]. Nonetheless, its possible mechanism of action remains fully elucidated.

Enterovirus 71 (EV71) is a non-enveloped, positive-sense single-stranded RNA virus in the family *Picornaviridae*, genus *Enterovirus*. The EV71 genomic encodes four structural proteins (VP1, VP3, VP0, VP4) and seven non-structural proteins (2A–2C, 3A–3D) [9]. Since polioviruses were eliminated from the gut by polio vaccination, EV71 has emerged as a prominent cause of hand, foot, and mouth disease (HFMD) and a public health concern in China [10,11]. However, there are currently no globally approved antiviral therapies or vaccines available for EV71. Outbreak control relies on non-pharmaceutical interventions, underscoring the need for new antiviral strategies.

The SH3 and cysteine-rich domain protein (Stac) protein family comprises three members (Stac 1, Stac 2, Stac 3), Stac1 and Stac2 are expressed in the central nervous system, Stac1 is also in the gastrointestinal tract, while Stac3 is mainly localized in skeletal muscle [12]. Stac1 positively modulates the surface expression of voltage-activated calcium channels (VGCCs), and regulating downstream signaling cascades that mediate various cellular functions [13].

In this study, we delineate a previously unrecognized mechanism by which LL-37 inhibits non-enveloped virus EV71. To our knowledge, this is the first evidence that an antimicrobial peptide can suppress a non-enveloped virus by engaging a host factor, Stac. Transcriptomic profiling identified *Stac* as a gene selectively upregulated by LL-37 but not by EV71 infection. We further show that LL-37 induces Stac expression, probably through the EGFR-ERK signaling axis, and that Stac functions as a negative regulator of EV71 replication. These findings support a host-directed antiviral mechanism for LL-37 and highlight a potential therapeutic strategy for controlling EV71 infection.

## 2. Materials and Methods

### 2.1. Cell Culture, Viruses, and Materials

Caco-2 (Chinese Academy of Sciences, Shanghai, China), RD (BNCC, Zhengzhou, China), Vero (kindly gifted by Prof. Aihua Zheng), 293T (ATCC, Manassas, Virginia, USA) cells were cultured in DMEM (Gibco, Beijing, China) with 10% (*v*/*v*) fetal bovine serum (Gibco), 1% penicillin/streptomycin (Gibco) in a humidified atmosphere of 5% CO_2_ at 37 °C. EV71 viruses (MN171486.1) were propagated in RD cells. Manipulations were performed in a BSL-2 laboratory. LL-37 (Selleck, Houston, TX, USA) and Scramble LL-37 (scr, TargetMol, Boston, MA, USA) were dissolved in sterile water.

### 2.2. Cell Viability

Caco-2 cells seeded in 96-well plates were incubated with serial dilutions of LL-37 and scr for 24 h. Cell viability was determined using the Cell Counting Kit-8 (CCK-8, Vazyme, Nanjing, China). The absorbance was measured at a 450 nm wavelength.

### 2.3. Reverse Transcription-Quantitative Polymerase Chain Reaction (RT-qPCR)

Total RNA was extracted using the Trizol (Invitrogen, Carlsbad, CA, USA) and reverse transcribed using HiScript III 1st Strand cDNA Synthesis Kit (Vazyme). The qPCR was performed using AceQ Universal SYBR qPCR Master Mix (Vazyme). Gene expressions were normalized to cyclophilin. Expressions of the target gene were determined using the 2^−ΔΔ*C*t^ method. Primer sequences are listed in the Supplementary Table S1.

### 2.4. Viral Plaque Assay

This assay used a confluent monolayer of Vero cells plated in 24-well plates. Viral samples were subjected to serial dilutions and added to each well. Plates were incubated for 2 h, washed, and overlaid with 1% CMC (Sigma, St. Louis, MO, USA). Plaques were visible in the next 3–6 days. Cells were fixed with 10% formaldehyde solution and stained with 0.1% crystal violet (Solarbio, Beijing, China). 

### 2.5. Western Blot

Cells were lysed in RIPA buffer with protease inhibitor (Roche, Mannheim, Germany) and phosphatase inhibitor (Selleck). Proteins were separated by sodium dodecyl sulfate-polyacrylamide gel electrophoresis and transferred to PVDF membranes. Primary Antibodies used include anti-β-actin (Sigma), anti-Entervirus71 3C (GeneTex, Irvine, CA, USA), anti-GAPDH (Cell Signaling Technology, Danvers, MA, USA), p-ERK1/2 (Abmart, Shanghai, China), and ERK1/2 (Cell Signaling Technology). Band quantifications were quantified using *ImageJ* (v1.8.0.345).

### 2.6. RNA Sequencing (RNA-Seq)

Total RNA was sent to Novogene (Beijing, China) for the construction of cDNA libraries and transcriptomic sequencing (Illumina NovaSeq X-plus, Illumina, San Diego, CA, USA). Differential expression analysis was performed using the DESeq2 R package (1.20.0). Differentially expressed genes (DEGs) were defined with the thresholds of |log_2_(foldchange)| > 0.263 and *p*-value < 0.05.

### 2.7. Stac Knockdown

The shRNA for Stac (shStac) was synthesized by Tianyihuiyuan (Beijing, China). Sequences of shStac were listed in the Supplementary Table S2. The shStac oligonucleotides were annealed to form double-stranded DNA and cloned into the pLKO.1 plasmid via AgeI-EcoRI restriction sites. Empty pLKO.1 plasmid served as the control. The plasmids were transfected into 293T cells together with psPAX2 and pMD2.G using Polyethylenimine (Polyscience, Warrington, PA, USA). Stable Caco-2 cell lines were established by lentiviral infection followed by puromycin selection.

### 2.8. Flag-Tagged Stac Overexpression

The Flag-tagged Stac fragment was amplified using the pCDH-Stac (GeneScript, Nanjing, China) as a template. Homologous recombination between the purified Flag-Stac fragment and linearised pCAG-KOZAK-N-FLAG-DHX9 plasmid was performed using the ClonExpress II One Step Cloning Kit (Vazyme). Cells were transiently transfected using Lipofectamine™ 2000 Transfection Reagent (Invitrogen) for 24 h, followed by EV71 infection at a multiplicity of infection (MOI) value of 0.001. Cell samples were collected at 24 h post-infection (hpi).

### 2.9. Molecular Docking and Prediction of Potential Transcription Factors (TFs)

Structures of LL-37 (PDB:2K6O), Formyl peptide receptor 2 (FPR2, PDB:8Y62), and EGFR (PDB:7T4I and 5XWD) were selected for docking. Molecular docking was performed using the ClusPro Server (https://cluspro.org, accessed on 25 July 2025)., and the results were visualized using 3D Protein Imaging (https://3dproteinimaging.com, accessed on 8 September 2025). The binding affinity and stability were predicted using the separated binding energy per unit interface area (dG_separated/dSASA × 100, Rosetta 2024.09) [14] and packing statistics (packstat) [15].

The 2000 bp sequence upstream of ATG was extracted from the transcription start site of the *STAC1* gene sequence as the potential promoter region. The promoter sequence was submitted to the JASPAR database (https://jaspar.genereg.net, accessed on 17 September 2025) and predicted putative transcription factor binding sites using the JASPAR CORE set.

### 2.10. Statistical Analysis

Comparisons between two groups were made using unpaired *t*-tests, and comparisons among multiple groups were made using one- or two-way ANOVA, followed by Tukey’s or Dunnett’s post hoc tests. Statistical analysis was performed in GraphPad Prism (v10.6.0) Data were shown as mean ± standard deviation, where ns, *, **, ***, **** represent *p* > 0.05, *p* < 0.05, *p* < 0.01, *p* < 0.001, *p* < 0.0001, respectively.

## 3. Results

### 3.1. LL-37 Treatment Inhibits the Intracellular EV71 Replication in Caco-2 Cells

To determine a non-cytotoxic concentration range, we assessed Caco-2 cell viability after LL-37 or scr treatment. LL-37 exhibited no significant cytotoxicity at concentrations from 1 to 20 μg/mL, while cell viability was reduced by approximately 7% at 50 μg/mL (Supplementary Figure S1). Therefore, concentrations of 1–20 μg/mL were selected for subsequent experiments.

**Figure 1:**
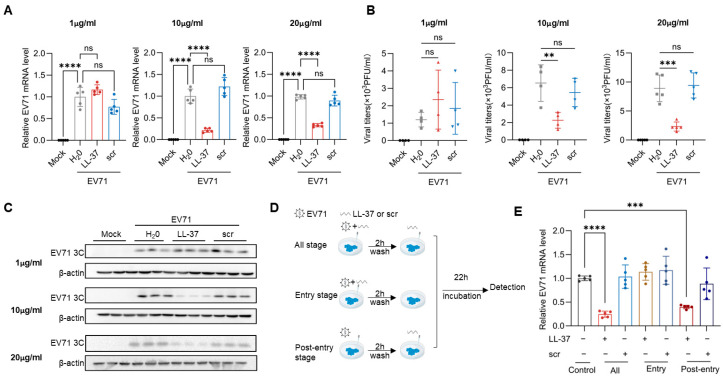
LL-37 dose-dependently inhibited Enterovirus 71 (EV71) infection in CaCo-2 cells. Caco-2 cells were treated with EV71 (MOI = 1) with sterile H_2_0, LL-37, or scramble LL-37 (scr) (1, 10, 20 μg/mL) for 24 h. Mock-infected group served as a negative control. The relative intracellular EV71 mRNA levels (**A**), infectious viral particles (**B**), and EV71 3C levels (**C**) were detected by RT-qPCR, PFU assay, and Western blot. (**D**) Schematic of the time-of-addition assay. LL-37 or scr (10 μg/mL) was administered to EV71-infected cells (MOI = 1) at distinct stages of viral infection. EV71-infected cells (untreated) served as positive controls. (**E**) Relative EV71 mRNA levels in each group were evaluated by RT-qPCR. Data were shown as mean ± standard deviation, where ns, **, ***, **** represent *p* > 0.05, *p* < 0.01, *p* < 0.001, *p* < 0.0001, respectively. Data were representative of independent biological replicates.

To examine the antiviral effects of LL-37, Caco-2 cells were treated with LL-37 or scr while infected with EV71. At 24 hpi, intracellular EV71 mRNA levels, viral titers, and EV71 3C protein expressions were analyzed by RT-qPCR, plaque-forming units (PFU) assays, and Western blot. LL-37, but not the scr, at concentrations of 10 and 20 μg/mL (but not 1 μg/mL), significantly reduced intracellular viral mRNA, viral titer, and 3C protein levels compared to the EV71-infected control (Figure 1A–C). Furthermore, LL-37 treatment efficiently reduced viral mRNA levels in the post-entry and all-stage assay, but not in the entry assay (Figure 1D,E). These data demonstrate that LL-37 inhibits EV71 infection in a dose-dependent manner, primarily by targeting a post-entry stage of the viral life cycle.

### 3.2. Stac Is an Important Factor for LL-37 Inhibiting EV71 Replication

**Figure 2:**
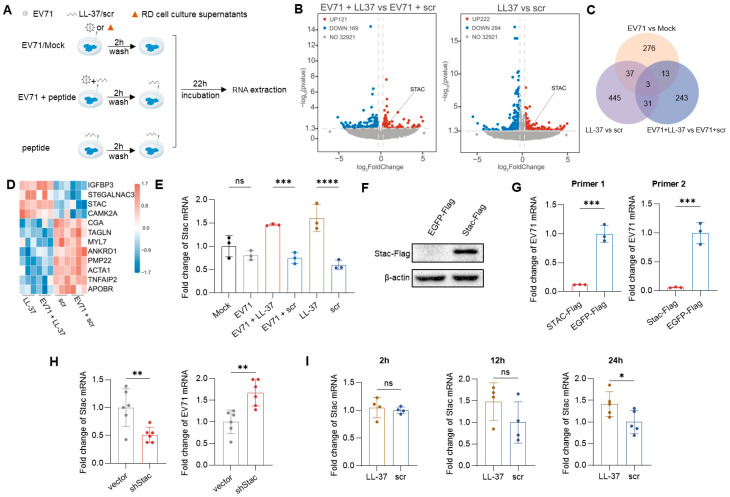
Stac suppressed EV71 infection in Caco-2 cells. (**A**) Schematic of the treatment procedure in transcriptomic experiments (EV71 vs. Mock, EV71 + LL-37 vs. EV71 + scr, LL-37 vs. scr) (*n* = 3). (**B**) Volcano plots of differentially expressed genes (DEGs) in the comparison group analysis (EV71 + LL-37 vs. EV71 + scr, LL-37 vs. scr). The dashed line indicates a threshold of significant magnitude at |log_2_(foldchange)| > 0.263 and *p*-value < 0.05. (**C**) Venn diagrams of DEGs. (**D**) Heatmap of 12 protein-coding genes overlapping in “EV71 + LL-37 vs. EV71 + scr” and “LL-37 vs. scr” (not in “EV71 vs. Mock”). (**E**) Validation of Stac expression (*n* = 3). Flag-tagged Stac proteins (**F**) and EV71 mRNA levels (**G**) were detected by Western blot and RT-qPCR with two primer pairs (*n* = 3, MOI = 0.001). (**H**) Relative Stac and EV71 mRNA levels in vector-control and shStac stable cell lines (*n* = 6, MOI = 0.1). (**I**) Stac expressions were detected at 2, 12, and 24 h post-peptide treatment by RT-qPCR (*n* = 4). Data were shown as mean ± standard deviation, where ns, *, **, ***, **** represent *p* > 0.05, *p* < 0.05, *p* < 0.01, *p* < 0.001, *p* < 0.0001, respectively. Data were representative of independent biological replicates.

To obtain insights into the mechanism by which LL-37 inhibited non-enveloped virus replication, Caco-2 cells were treated with LL-37 or scr, with and without EV71 infection (EV71 + LL-37 versus EV71 + scr, LL-37 versus scr). A comparison of EV71-infected and uninfected cells (EV71 versus Mock) was also conducted. RNA-seq was then performed (Figure 2A). The EV71 infection and antiviral efficacy of LL-37 were confirmed (Supplementary Figure S2). Based on *p* < 0.05 and |log_2_ foldchange| > 0.263, 1048 DEGs were identified by comparison analysis (Figure 2B, Supplementary Figure S3). The Venn diagram revealed 31 DGEs overlapped in “EV71 + LL-37 versus EV71 + scr” and “LL-37 versus scr” comparisons, with no overlap in “EV71 vs. Mock” comparison (Figure 2C). Based on a cutoff value of |foldchange| > 1.5, expressions of 12 protein-coding genes *(STAC*, *IGFBP3*, *ST6GALNAC3*, *CAMK2A*, *CGA*, *TAGLN*, *MYL7*, *ANKRD1*, *PMP22*, *ACTA1*, *TNFAIP2*, *APOBR*) were illustrated in the heatmap (Figure 2D) and confirmed by qPCR (Figure 2E, Supplementary Figure S4). Among them, *Stac* mRNA expression was 1.8-fold higher in the EV71 + LL-37 group and 2.77-fold higher in the LL-37 group compared to their respective controls, with no significant difference between the EV71 and the Mock groups (Figure 2E). These results indicate that LL-37 specifically upregulates the Stac expression irrespective of EV71 infection, suggesting that the Stac may contribute to the protective role of LL-37 following viral challenge.

To investigate the function of Stac during EV71 infection, Caco-2 cells were transiently transfected with plasmids expressing Stac-Flag and EGFP-Flag, and subsequently infected with EV71. Western blot analysis confirmed the overexpression of Stac protein (Figure 2F). The relative level of EV71 mRNA was assayed using two independent primer pairs, and both confirmed reduced viral mRNA with Stac overexpression. Specifically, viral mRNA in the Stac-Flag was undetectable using primer pair 2 and was 0.18-fold (a 92% reduction) of the EGFP-Flag control with primer pair 1 (Figure 2G). To further validate the role of Stac, we used RNA interference to knock down *Stac* expression in Caco-2 cells. The knockdown efficiency was confirmed using RT-qPCR (Figure 2H left). Stac knockdown promoted viral replication, as evidenced by a significant upregulation of EV7l mRNA level in the shStac group (Figure 2H right). Collectively, these data demonstrate that Stac suppresses viral replication.

To validate the effect of LL-37 on Stac expression, Caco-2 cells were incubated with 10 μg/mL LL-37 or scr for 2, 12, and 24 h. LL-37 treatment did not significantly affect *Stac* expression at the 2 and 12 h time points, although a slight trend toward an increase at the 12-h time point. In accordance with transcriptomic results, LL-37 treatment significantly increased the *Stac* mRNA level at the 24 h time point (Figure 2F).

### 3.3. Upregulation of the Stac by LL-37 via the EGFR-ERK Pathway

**Figure 3:**
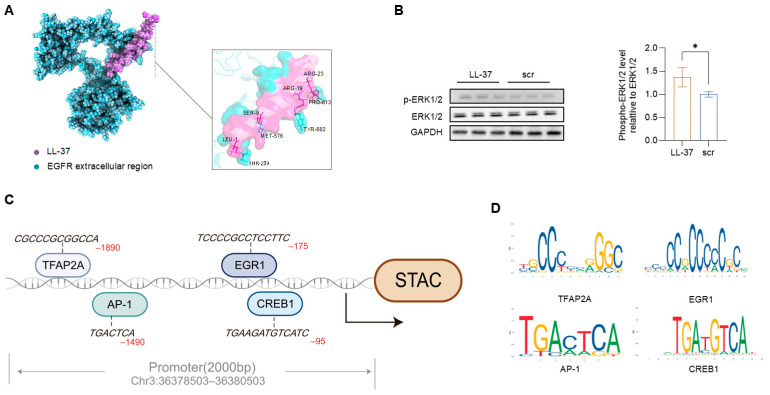
LL-37 activated the EGFR–ERK signaling pathway and promoted Stac transcriptional. (**A**) Molecular docking diagram showed the predicted binding pose of LL-37 (purple) with the extracellular region of EGFR (blue). (**B**) Caco-2 cells were treated with peptides (10 μg/mL of LL-37 or scr) for 24 h. Phosphorylated ERK1/2, total ERK1/2, and GAPDH were detected by Western blot. The ratios of p-ERK1/2 to ERK1/2 were analyzed by ImageJ (*n* = 3). (**C**) The binding of transcription factors (TFAP2A, AP-1, EGR1, CREB1) to the Stac promoter induced gene expression. (**D**) Transcription factors’ binding motifs. Data were shown as mean ± standard deviation, where * represent *p* < 0.05. Data were representative of independent biological replicates.

LL-37 interacts with plasma membrane receptors, including GPCRs, RTKs [5], and stimulates EGFR phosphorylation [16]. We performed molecular docking by calculating the binding energies and stability of LL-37 with FPR2, the intracellular and extracellular region of EGFR (Supplementary Table S2). To explore potential receptor engagement, we performed in silico docking analysis using published structures of LL-37 and EGFR. The modeling predicted structural compatibility between LL-37 and the extracellular domain of EGFR (Figure 3A). LL-37 treatment significantly increased ERK1/2 phosphorylation (Figure 3B), whereas PI3K phosphorylation was not affected (Supplementary Figure S5), supporting preferential activation of the EGFR–ERK axis under our experimental conditions.

TFs activated by the phosphorylation of ERK1/2 are critical regulators of cell proliferation, apoptosis, and intracellular calcium homeostasis [17,18]. JASPAR analysis identified high-affinity TF binding motifs on the Stac promoter. Candidate TF included CREB1, AP-1 (Fos/Jun), EGR1, and TFAP2A (Figure 3C,D). These findings suggest that the TFs from the ERK pathway regulate Stac expression. 

## 4. Discussion

LL-37 is a natural human antimicrobial peptide with broad-spectrum antiviral activity, through the mechanism of action can vary depending on the virus. In our study, we uncovered a novel host-directed mechanism for LL-37’s antiviral effect against a non-enveloped virus. We found that LL-37 inhibited V71 infection at a post-entry stage by upregulating the host gene through the ERK-EGFR signaling pathway.

Previous studies have elucidated several antiviral mechanisms of LL-37, especially against enveloped viruses. A primary mode of action against enveloped viruses is direct disruption of the viral envelope. LL-37 integrates into the viral envelope, causing structural modifications that inactivate the virus. For example, LL-37 treatment disrupted the integrity of SARS-CoV-2 viral membrane [19] and induced aggregation of Venezuelan equine encephalitis virus [20]. Furthermore, using the bacteriophage “Phi6” as a surrogate for enveloped viruses, LL-37 separated the envelope from the nucleocapsid, inactivating the virus [21]. LL-37 also inhibits viral fusion with the host membrane. For instance, LL-37 blocked Ebola virus (EBOV) entry by impairing cathepsin B-mediated processing of EBOV glycoprotein [22]. Collectively, these studies indicate that LL-37 often acts at early stages of infection to neutralize enveloped viruses.

However, for non-enveloped viruses like EV71, LL-37 appears to rely on alternative strategies. LL-37 failed to inactivate Coxsackievirus B3 when pre-incubated with virions [23]. Instead, it directly interacted with host exosomal heat shock protein 60, restricting viral infection established by exosomes. This suggests that LL-37’s antiviral action against non-enveloped viruses may predominantly occur via modulation of host cell processes rather than direct virion destruction. Our current findings align with the concept, as we demonstrate that LL-37 combats EV71 by upregulating a protective host gene without directly targeting the virus particle.

The Stac protein promotes surface functional expression of VGCCs and suppresses calcium-dependent inactivation, thereby regulating Ca^2+^ influx in nerve and skeletal muscle cells [24,25]. Our transcription analysis reveals that LL-37 robustly upregulates the Stac expression, irrespective of viral infection. LL-37 may indirectly regulate diverse Ca^2+^-dependent processes, including survival, apoptosis, autophagy, and transcriptional regulation [26], through the Stac protein. Ca^2+^ signaling is critically exploited by viruses to facilitate infection, while also modulating innate immune responses. For example, influenza A viruses manipulated intracellular Ca^2+^ dynamics to establish replication [27]. Wang et al. also demonstrated that wounding-triggered Ca^2+^ signaling induced RNA interference to combat viral infection [28]. We found Stac knockdown enhanced EV71 infection, whereas its overexpression diminished it, suggesting a preventive role for Stac during viral infection. The regulation of Stac expression represents a novel, broadly applicable mechanism underlying LL-37’s antiviral effects.

The antiviral effects of LL-37 against EV71 are indirect interactions with virions. Nuclear magnetic resonance (NMR) titration and immunoprecipitation assays detected no new binding peaks in the mixture of LL-37 and EV71 virion [29]. Pre-incubation of LL-37 and EV71 did not enhance EV71 inhibition, and although LL-37 reduced the binding of EV71 to U251cells [30]. Similarly, it mainly exerted its effects during the post-entry stage in Caco-2 cells. A general understanding of LL-37’s antiviral mechanism is that it regulates the host’s immune response, for example, LL-37 enhanced basal virus-induced IRF3 activation and IFN-β expression and suppressed pro-inflammatory cytokine IL-6 via the p38 MAPK pathway [30]. In our study, using an unbiased transcriptomic approach, we identify Stac as a previously unrecognized gene that is upregulated by LL-37. This discovery adds a new dimension to the known immunomodulatory and host-directed activities of LL-37, highlighting a mechanism that does not directly involve canonical antiviral cytokines but instead leverages a host cellular protein to impede viral replication.

Previous studies have shown that LL-37 can activate EGFR signaling in epithelial systems, often through GPCR/MMP-dependent transactivation mechanisms [31,32]. In these models, LL-37 promotes the shedding of EGFR ligands such as HB-EGF, which subsequently bind to and activate the receptor. LL-37 dose- and time-dependently induced EGFR and downstream ERK phosphorylation in human corneal epithelial cells [31], and also activated EGFR and ERK signaling in keratinocytes [33]. Our findings are consistent with the possibility that LL-37 activates EGFR through a similar indirect mechanism. In the present study, LL-37 treatment resulted in ERK1/2 phosphorylation and time-dependent induction of Stac expression. While structural modeling suggests spatial compatibility between LL-37 and the extracellular region of EGFR, we do not interpret this as evidence of direct receptor binding. Further biochemical and structural studies would be required to define the precise mode of receptor activation.

LL-37 likely employs multiple complementary routes to exert its antiviral effects. The precise mechanism by which Stac interferes with viral replication remains to be clarified. It is conceivable that Stac, through its influence on calcium channel function and downstream signaling, alters cellular conditions in ways that inhibit viral RNA replication or protein synthesis. Alternatively, Stac might counteract EV71-induced pathological changes, such as calcium imbalance or cell death pathways, that would otherwise facilitate viral propagation. Further studies are needed to delineate the downstream effectors and pathways by which Stac exerts its antiviral function, as well as to determine whether other host factors identified in our transcriptomic analysis also contribute to LL-37’s antiviral activity.

## 5. Conclusions

In conclusion, this study demonstrates that the antimicrobial peptide LL-37 upregulates the expression of the host gene, Stac, by which mechanism it exerts antiviral effects in the post entry stage of the EV71 life cycle. Stac functions as a negative regulator of EV71 replication, and is selectively upregulated by LL-37 but not by EV71 infection. We further show that LL-37 induces Stac expression, probably through the EGFR-ERK signaling axis. These data elucidate a host-directed antiviral mechanism by which LL-37 inactivates non-envelope viruses, and show potential promise in combating viral infections.

## Data Availability

RNA sequencing data were deposited in the NCBI GEO database under the accession number GSE311775.

## Supplementary Materials

The following supporting information can be downloaded at: https://www.mdpi.com/article/10.3390/v18040442/s1, Figure S1: Cell viability. Cytotoxicity of LL-37 in Caco-2 cells was determined by the CCK8 assay. Cells were treated with increasing concentrations of LL-37 and scrLL-37 (1, 10, 20, and 50 μg/mL) for 24 h (*n* = 6). Figure S2: Validation of EV71 expression in transcriptomic samples. Figure S3: Volcano plots of DEGs in the comparison group “EV71 vs. Mock”. Figure S4: Validation of the expressions of genes (*CAMK2A*, *IGFBP3*, *ANKRD1*, *TAGLN*, *CGA*, *TNFAIP2*, *ACTA1*, *APOBR*, *MYLT*, *ST6GALNAC3* and *PMP22*) (*n* = 3). Figure S5: Phosphorylation of PI3K. Caco-2 cells were treated with peptides (10 μg/mL of LL-37 or scr) for 24 h. Phosphorylated PI3K, total PI3K, and GAPDH were detected by Western blot. The ratios of p-PI3K to PI3K were analyzed by ImageJ (*n* = 3 biological replicates). Table S1: Primer sequences; Table S2: Sequences of ShStac used in this study. Table S3: Molecular docking calculation scores of LL-37 and receptors.
