# Supplementary material for: LL-37 Inhibits EV71 Infection by Upregulating STAC via the EGFR-ERK Signaling Pathway
Source: Viruses. 2026 Apr 7;18(4):442. doi: 10.3390/v18040442 (PMC13120454; doi:10.3390/v18040442)
Supplement: Supplementary file 1 [file viruses-18-00442-s001.zip › Supplemengtary Materials from LL-37.pdf]

Supplementary Materials

LL-37 inhibits EV71 infection by upregulating STAC via the EGFR-ERK signaling pathway

Jiaqi Zhang [1,2,†], Hanlin Zhang [3,†], Yi Chen [1,2,†], Hanfei Liu [1,2], Shuhuang Peng [1,2], Jiwei Zhao [1,2], Zhe Luan [2], Yujian Zhang [3,4], Meng Dong [3], Wanzhu Jin [3,4,*] and Gang Sun [1,2,*]

1 Medical School of Chinese People's Liberation Army, Beijing 100853, China; jennyzjq@163.com (J.Z.); cydoctor26@163.com (Y.C.); liuhanfei0228@163.co(H.L.); psh8576@163.com (S.P.); zhaojiwei1005@163.com (J.Z.)
2 Department of Gastroenterology and Hepatology, The First Medical Center, Chinese PLA General Hospital, No. 28 Fuxing Road, Haidian District, Beijing 100853, China; luanzhe@foxmail.com
3 State Key Laboratory of Animal Biodiversity Conservation and Integrated Pest Management, Institute of Zoology, Chinese Academy of Sciences, Beijing 100101, China; zhanghanlin@ioz.ac.cn (H.Z.); zhangyujian2023@ioz.ac.cn (Y.Z.); dongmeng@ioz.ac.cn (M.D.)
4 College of Life Sciences, University of Chinese Academy of Sciences, Beijing 101408, China
* Correspondence: jinw@ioz.ac.cn (W.J.); sungang@301hospital.com.cn (G.S.); Tel.: +86-15010099771 (W.J.); +86-13501078679 (G.S.)
† These authors contributed equally to this work.

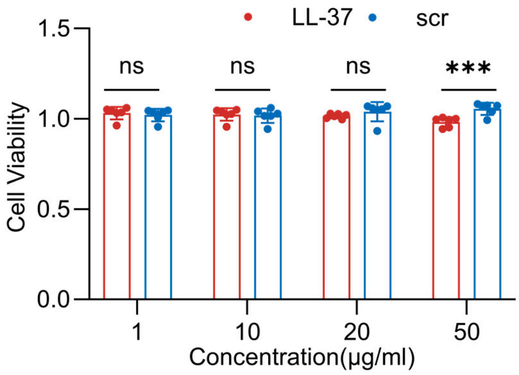

Supplement Figure S1. Cell viability. Cytotoxicity of LL-37 in Caco-2 cells was determined by the CCK8 assay. Cells were treated with increasing concentrations of LL-37 and scrLL-37(1, 10, 20, and 50μg/ml) for 24h (*n*=6).

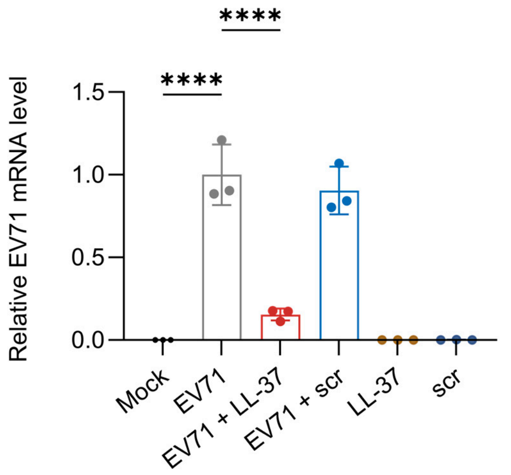

Supplement Figure S2. Validation of EV71 expression in transcriptomic samples.

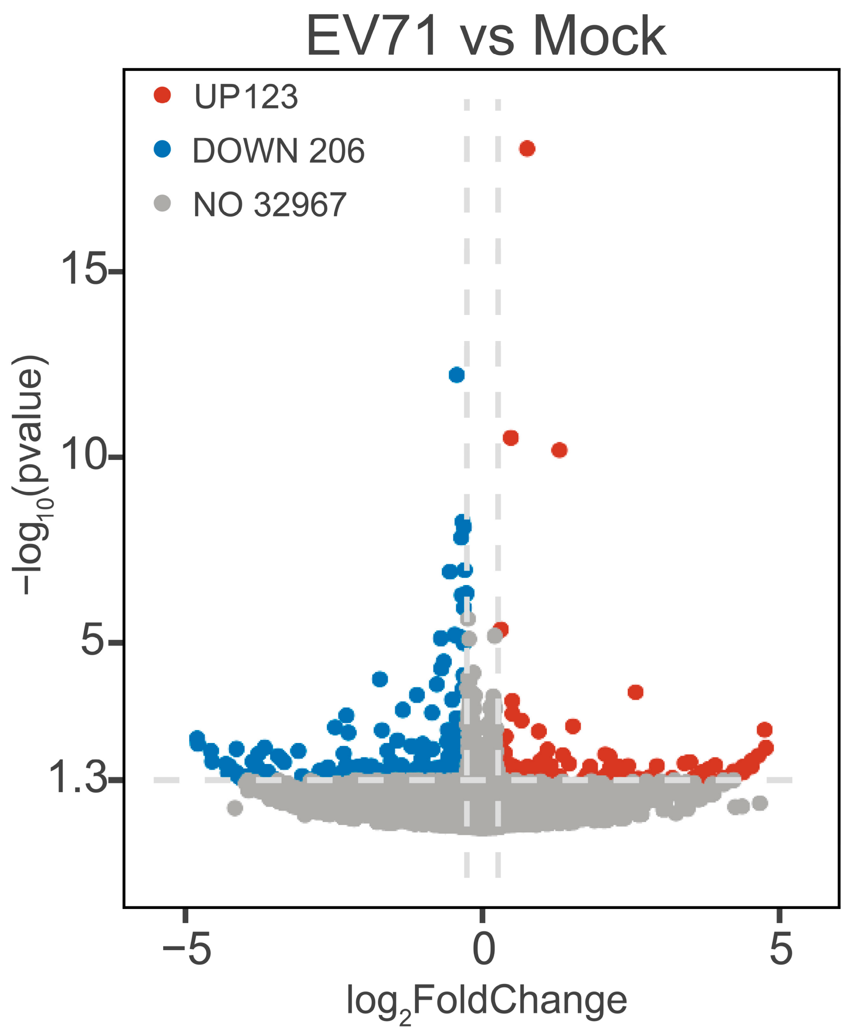

Supplement Figure S3. Volcano plots of DEGs in the comparison group "EV71 vs Mock".

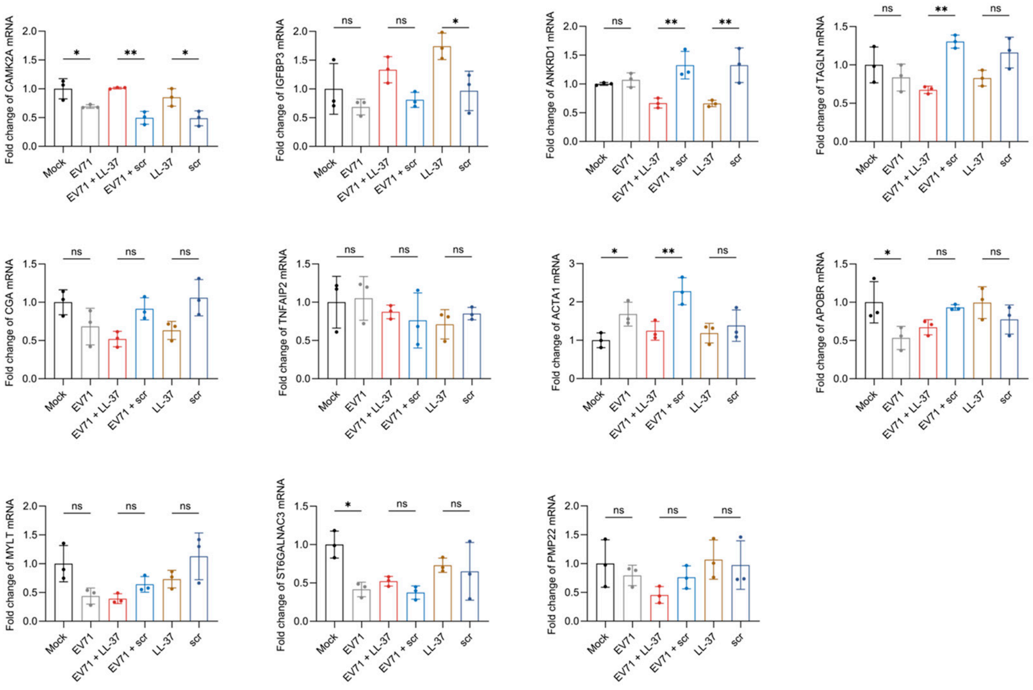

Supplement Figure S4. Validation of the expressions of genes(*CAMK2A, IGFBP3, ANKRD1, TAGLN, CGA, TNFAIP2, ACTA1, APOBR, MYLT, ST6GALNAC3* and *PMP22*) (*n*=3).

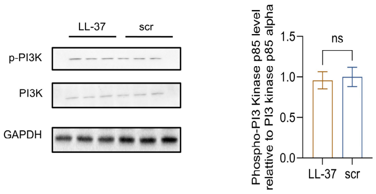

Supplement Figure S5. Phosphorylation of PI3K. Caco-2 cells were treated with peptides (10μg/ml of LL-37 or scr) for 24h. Phosphorylated PI3K, total PI3K, and GAPDH were detected by Western blot. The ratios of p-PI3K to PI3K were analyzed by ImageJ (*n* = 3 biological replicates).

Supplementary Table S1. Primer sequences

Genes	Forward primer	Reverse primer
EV71(primer1)	TATGGAGACGATGTGCTCGC	GGAAGGTCGCATTACCCCAA
EV71(primer2)	TGAGTGGCCTTCCTACTGCT	GCAGAACCCTGATCGGTAGA
Cyclophilin	CCGAGGAAAACCGTGTACTATTAG	TGCTGTCTTTGGGACCTTG
Stac	CAGCCAGGAATCCAAGCTCC	CCATGTGTGCTTGCAGTTTCA
CAMK2A	AAGAAAGTCCAGTTCCAGCGTTC	TTCCTGTTTCCGCACTTTGGTG
IGFBP3	CAGGAAATGCTAGTGAGTCGGA	CTCTACGGCAGGGACCATATTC
ACTA1	GGCATTCACGAGACCACCTAC	CGACATGACGTTGTTGGCATAC
ANKRD1	TCAACGCCAAAGACAGAGAAGG	AGATCCGCGCCATACATAATCA
TAGLN	TGGAGTGGATCATAGTGCAGTG	CACCAGCTTGCTCAGAATCACG
TNFAIP2	CTGACGAATTACAGGGCCAAT	TGCGTGAACCTCTTGAACAGT
APOBR	AGCTTTCTGGACACACAGACA	TGCCGAGGGAATCCAGT
CGA	AAAGCCCAGAGAAAGGAGCG	GCAGAAACACCGACAATGTGA
MYL7	GGGTGGTGAACAAGGATGAGT	CGAACATCTGCTCCACCTCAG
ST6GALNAC3	GCCTGCATCCTGAAGAGAAAGT	ATTTACAAGACGCACAACCAGC
PMP22	GGAAGAAGGGGTTACGCTGTTT	CACGATCCATTGGCTGACGA

Supplementary Table S2. Sequences of ShStac used in this study

Forward	CCGGCCAGCCAACTTTGTTCAGAGACTCGAGTCTCTGAACAAAGTTGGCTGGTTTTTG
Reverse	AATTCAAAAACCAGCCAACTTTGTTCAGAGACTCGAGTCTCTGAACAAAGTTGGCTGG

Supplementary Table S3. Molecular docking calculation scores of LL-37 and receptors.

Receptor	Ligand	dG_separated/dSASAx100 (< -1.5)	Packstat (>0.65)
FPR2(8Y62)	LL-37 (2K6O)	-3.476	0.596
EGFR(7T4I）	LL-37 (2K6O)	-3.133	0.613
EGFR(5xwd）	LL-37 (2K6O)	-2.868	0.659
