# Supplementary material for: LL-37 Inhibits EV71 Infection by Upregulating STAC via the EGFR-ERK Signaling Pathway
Source: Viruses. 2026 Apr 7;18(4):442. doi: 10.3390/v18040442 (PMC13120454; doi:10.3390/v18040442)

EV 71 for Fig 1C(1 μg/ml)

1μg/ml

EV71 3C, 21kd

β-actin, 42kd

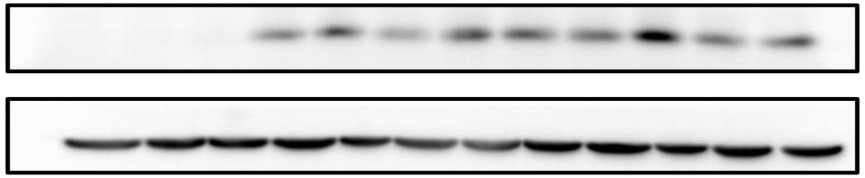

EV71 3C 21kd

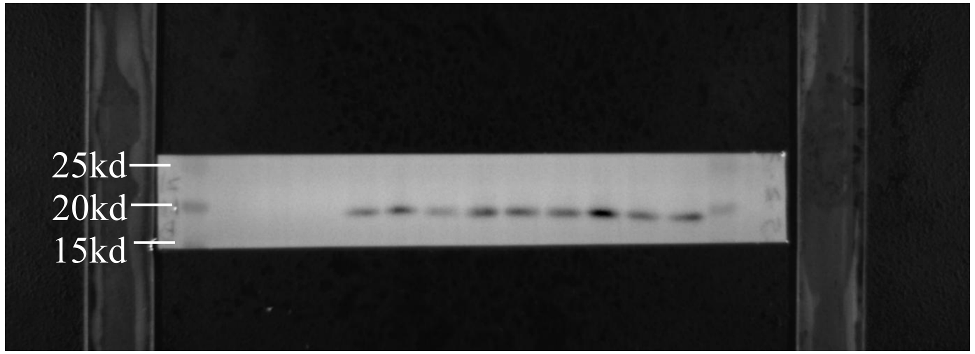

β-actin 42kd

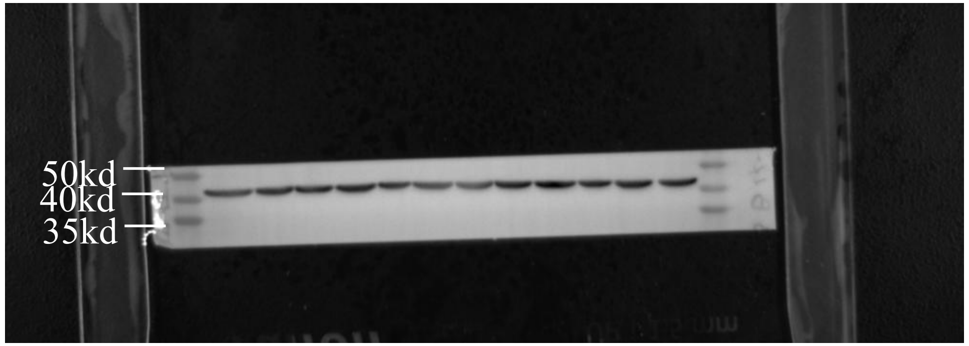

EV 71 for Fig 1C(10 μg/ml)

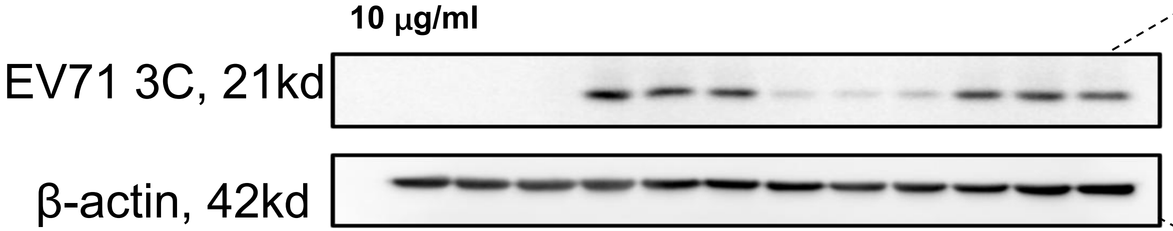

EV71 3C
21kd

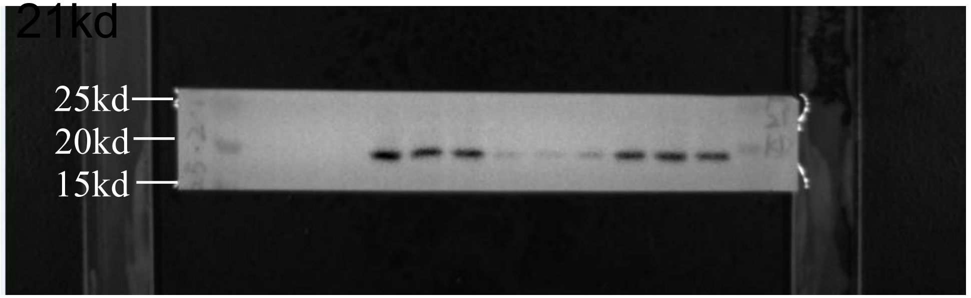

β-actin 42kd

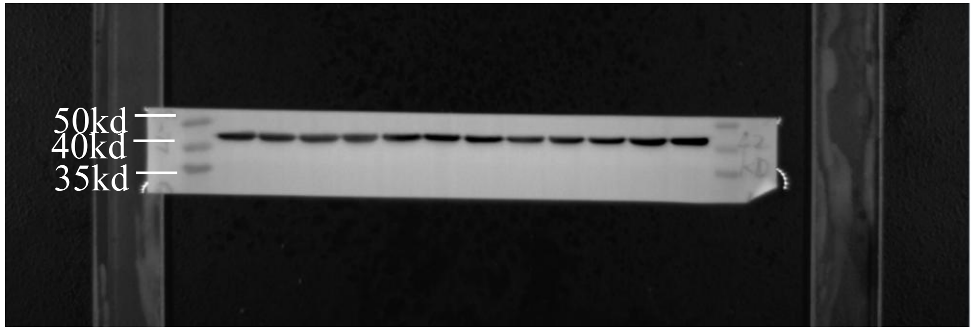

EV 71 for Fig 1C(20 μg/ml)

20 μg/ml

EV71 3C, 21kd

β-actin, 42kd

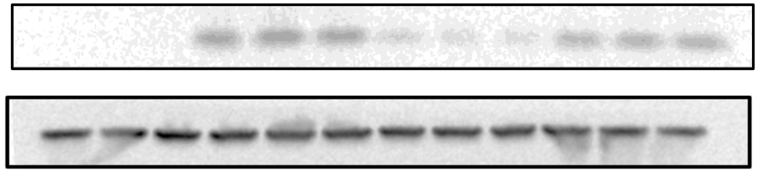

EV71 3C 21kd

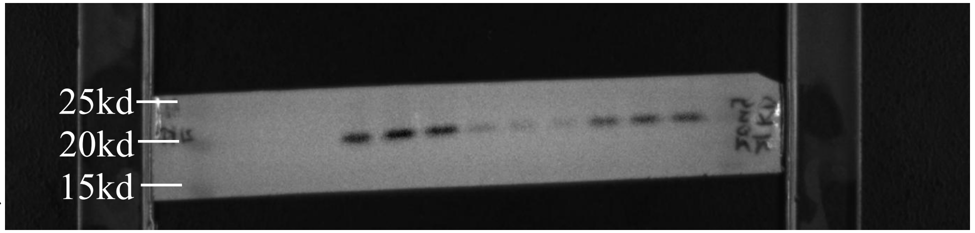

β-actin 42kd

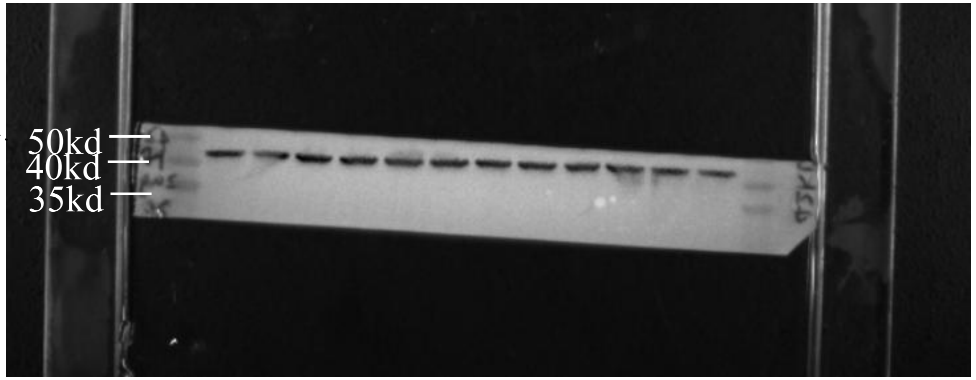

Stac for Fig 2F

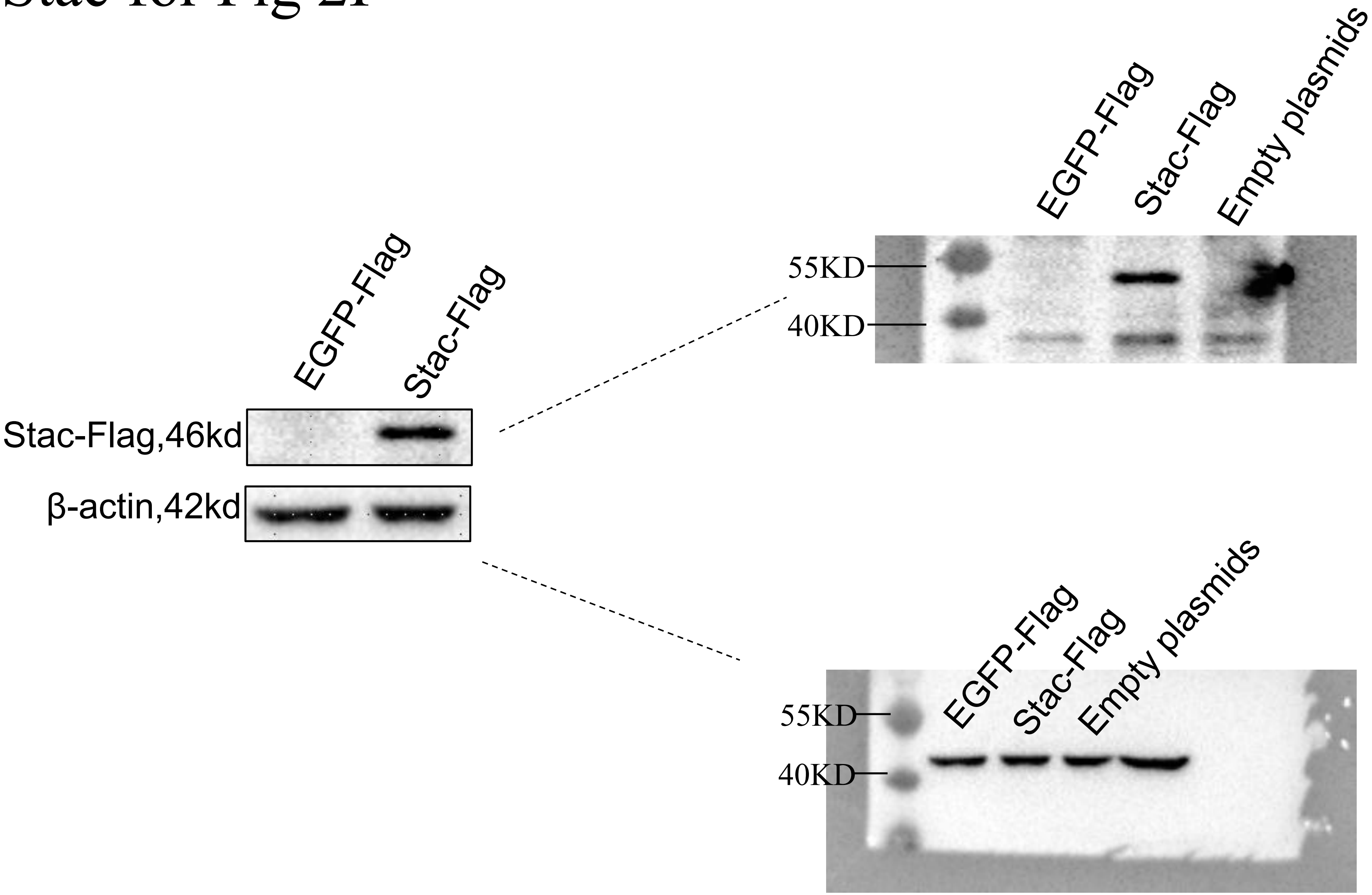

ERK for Fig 3B

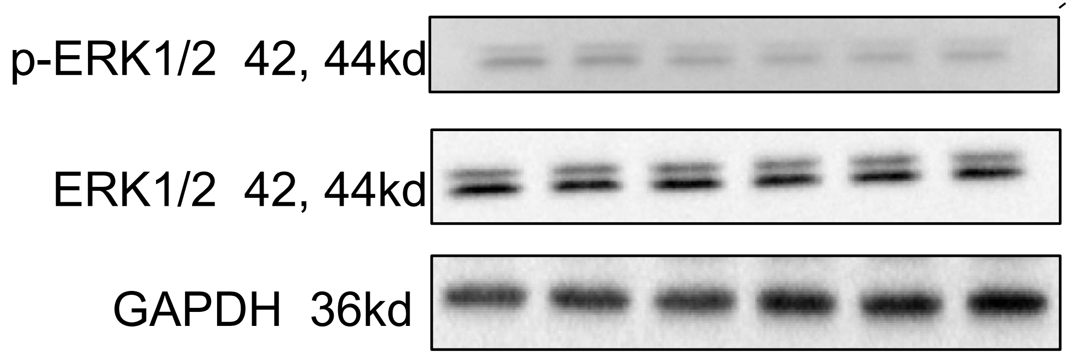

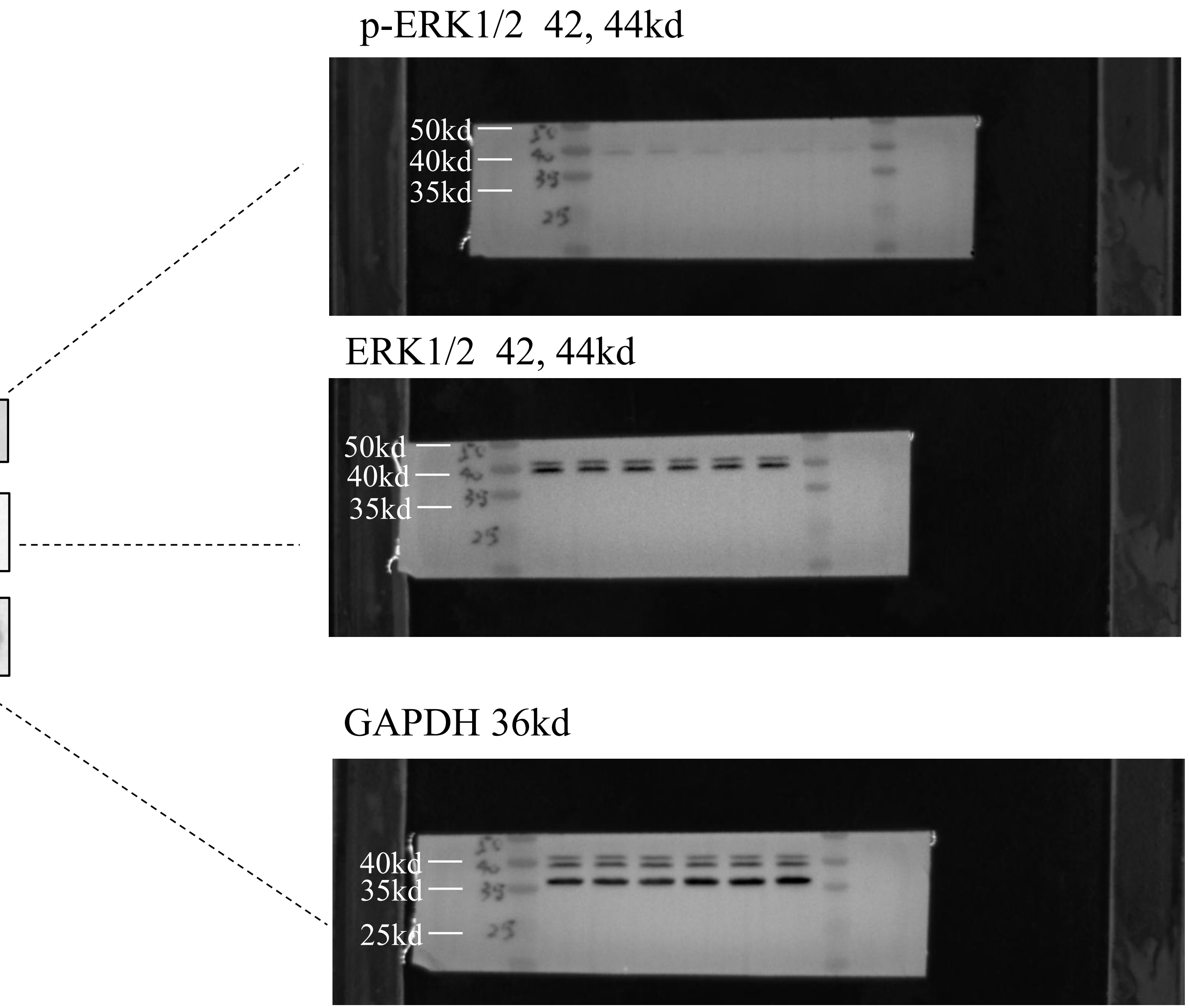

PI3K for Supplementary Fig2

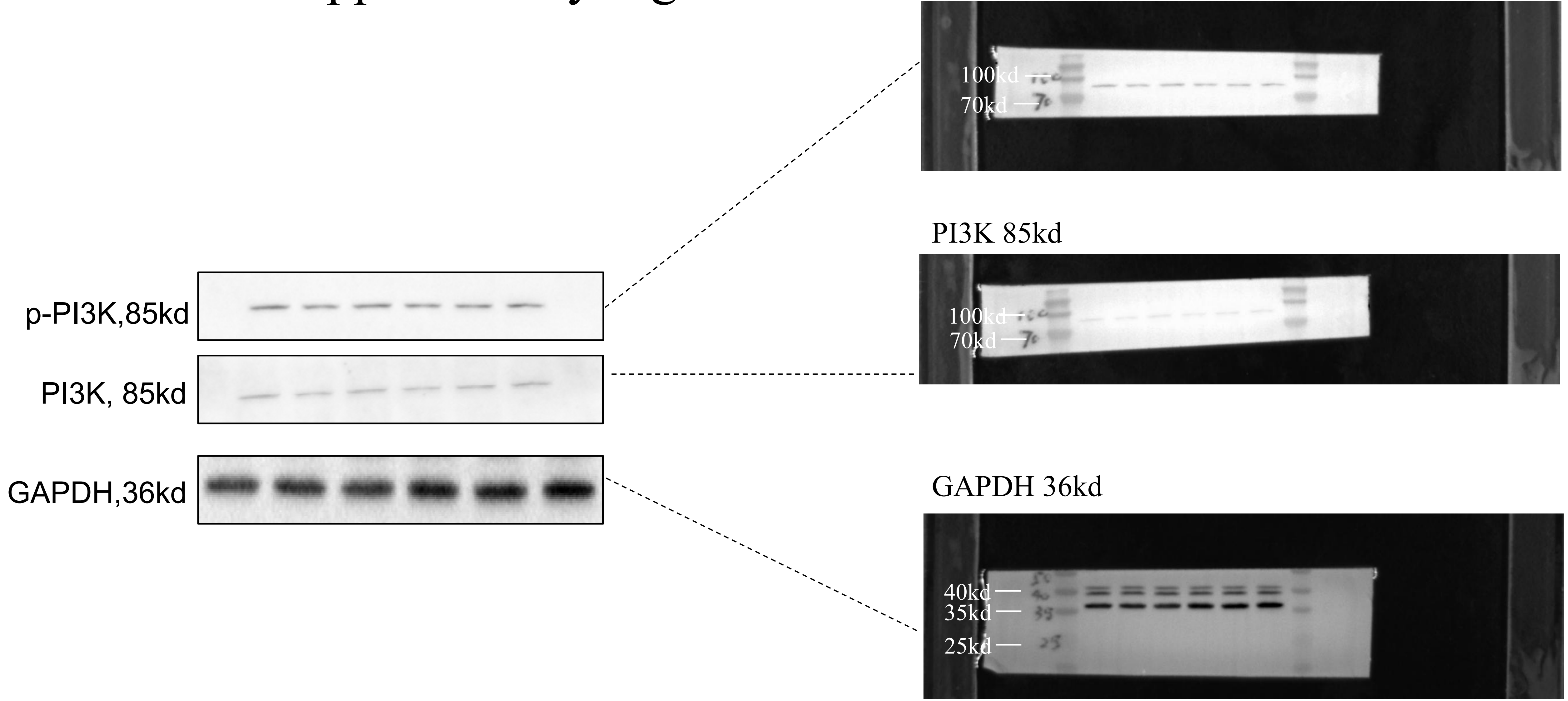

Supplement: Supplementary file 1 [file viruses-18-00442-s001.zip › WB .pdf]
