# Supplementary figures and images for: LL-37 Inhibits EV71 Infection by Upregulating STAC via the EGFR-ERK Signaling Pathway
Source: Viruses. 2026 Apr 7;18(4):442. doi: 10.3390/v18040442 (PMC13120454; doi:10.3390/v18040442)

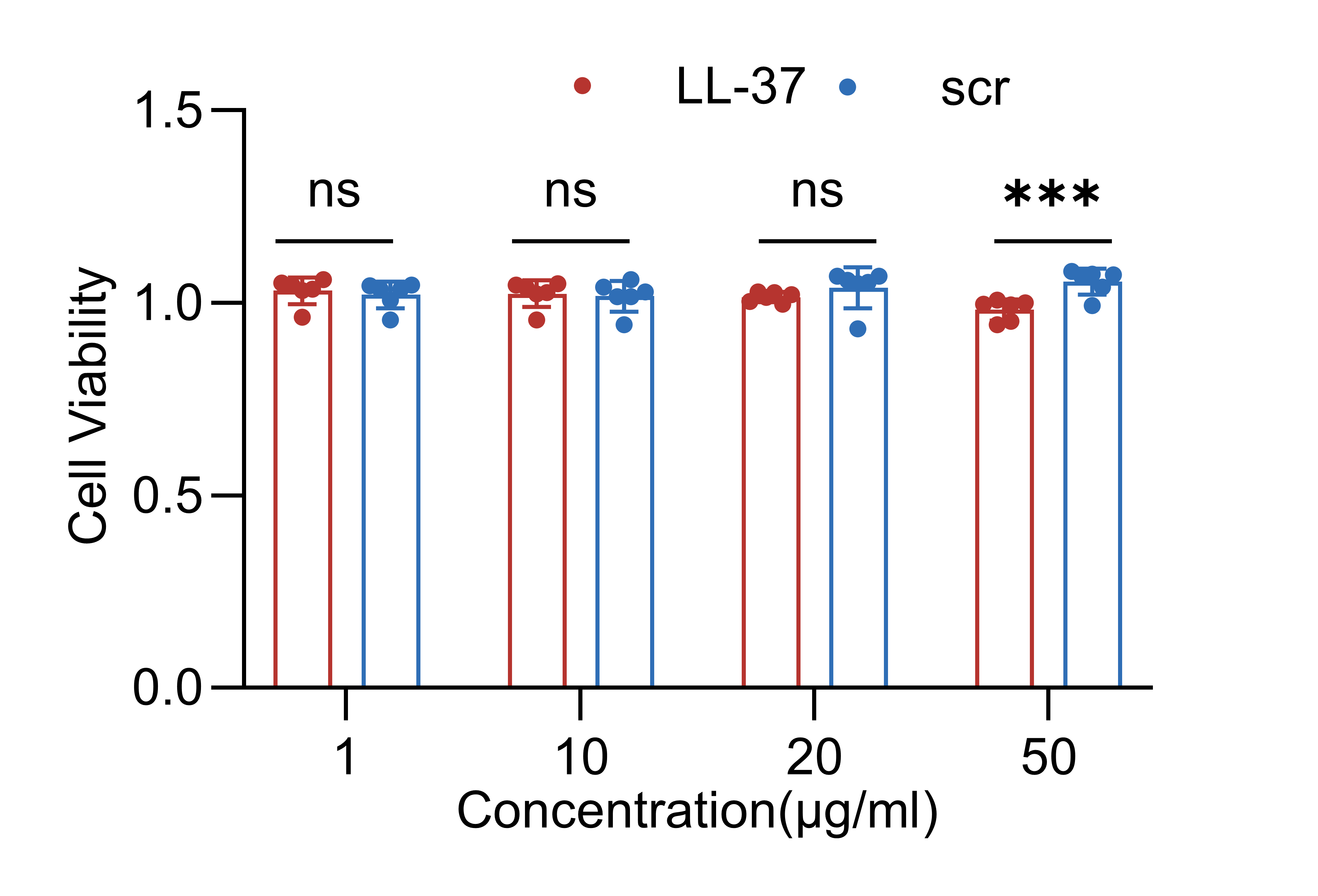

Supplement: Supplementary file 1 [file viruses-18-00442-s001.zip › Supplemengtary Figure S1.tif]

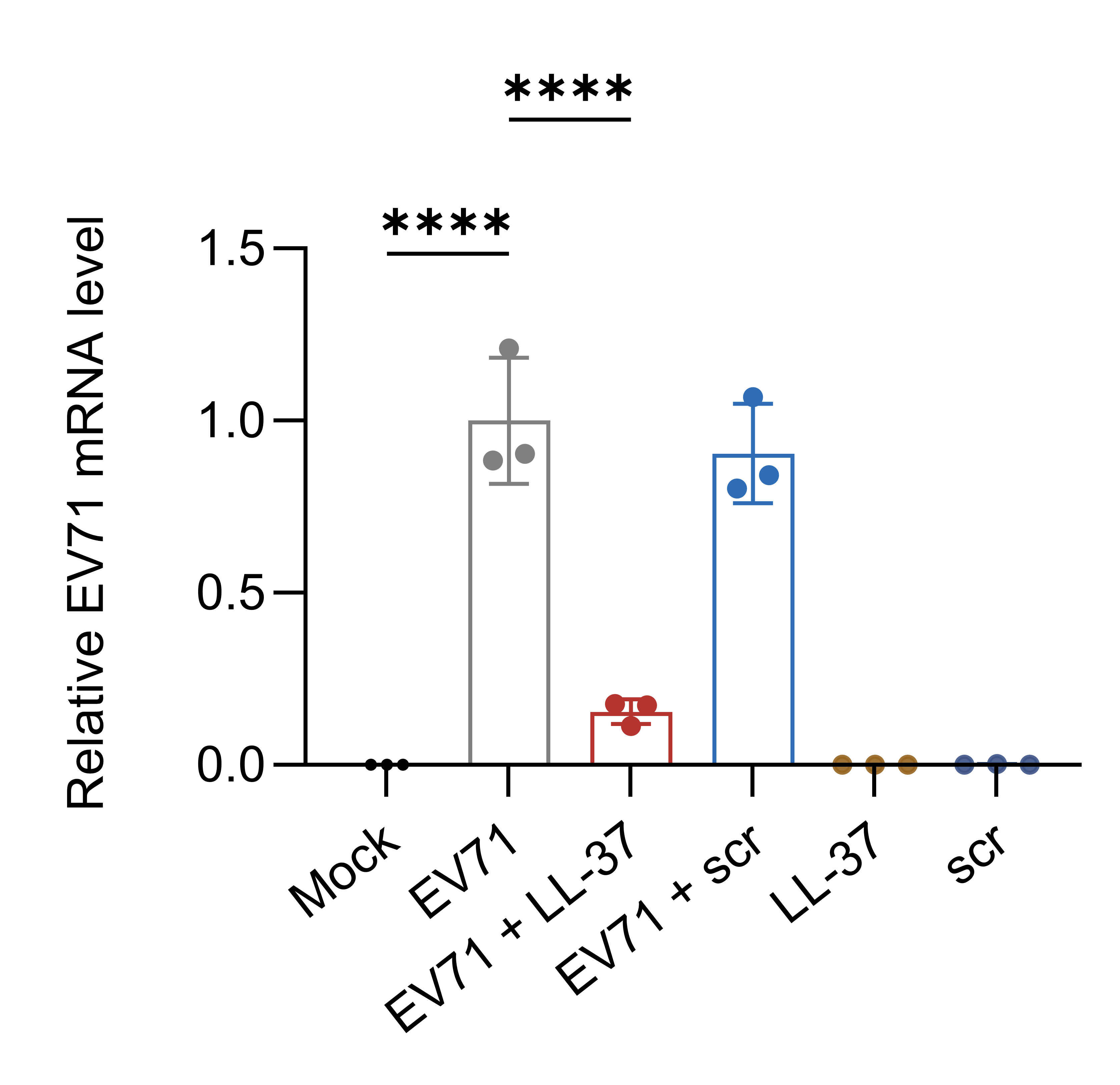

Supplement: Supplementary file 1 [file viruses-18-00442-s001.zip › Supplemengtary Figure S2.tif]

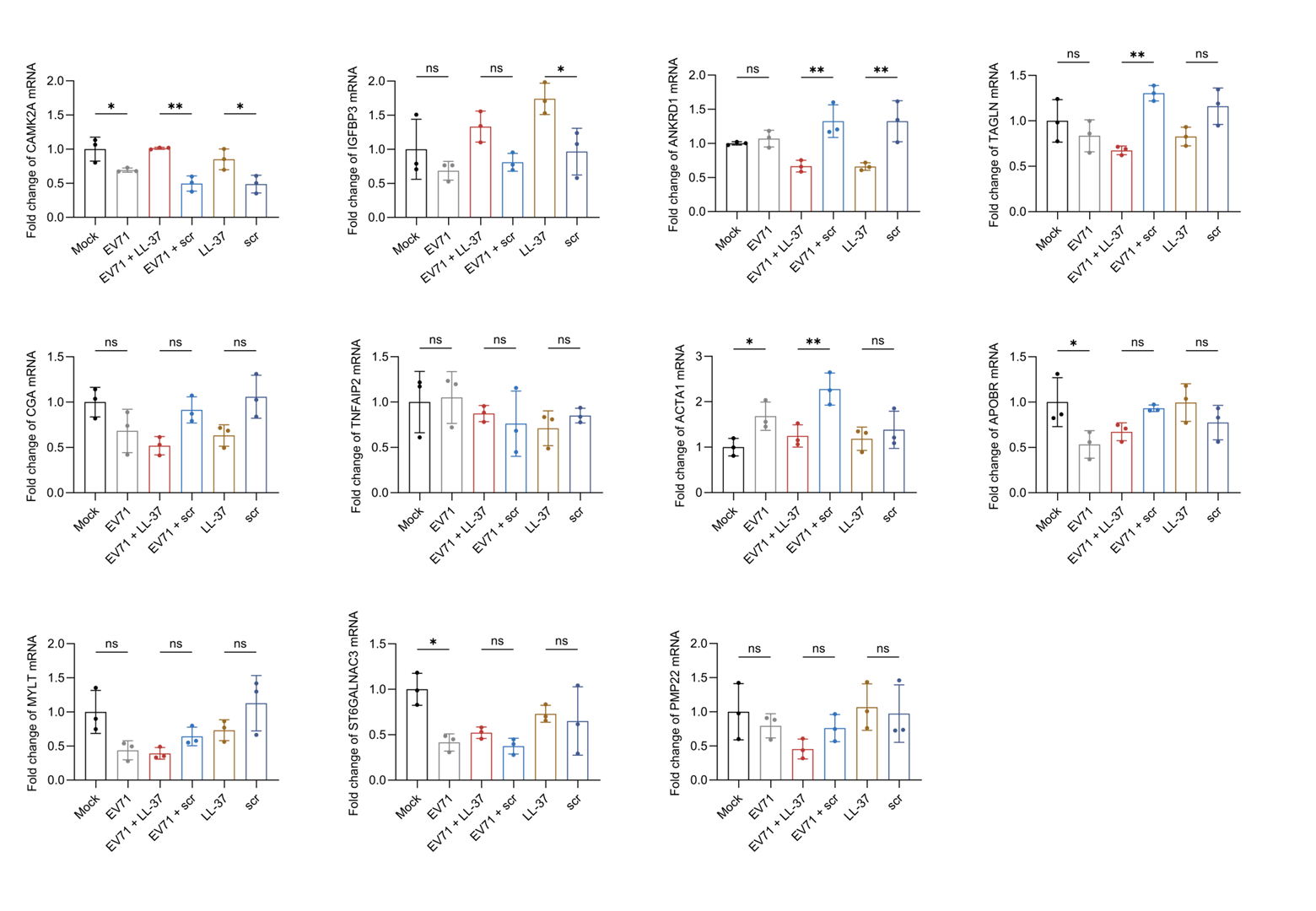

Supplement: Supplementary file 1 [file viruses-18-00442-s001.zip › Supplemengtary Figure S4.tiff]
